# Stability enhancement in a mAb and Fab coformulation

**DOI:** 10.1038/s41598-020-77989-w

**Published:** 2020-12-03

**Authors:** Hongyu Zhang, Paul A. Dalby

**Affiliations:** 1 Department of Biochemical Engineering, University College London, London, WC1E 6BT UK; 2 EPSRC Future Targeted Healthcare Manufacturing Hub, University College London, London, WC1E 6BT UK

**Keywords:** Biochemistry, Biological techniques, Biotechnology

## Abstract

Multiple therapeutic proteins can be combined into a single dose for synergistic targeting to multiple sites of action. Such proteins would be mixed in dose-specific ratios to provide the correct potency for each component, and yet the formulations must also preserve their activity and keep degradation to a minimum. Mixing different therapeutic proteins could adversely affect their stability, and reduce the shelf life of each individual component, making the control of such products very challenging. In this study, a therapeutic monoclonal antibody and a related Fab fragment, were combined to investigate the impact of coformulation on their degradation kinetics. Under mildly destabilizing conditions, these proteins were found to protect each other from degradation. The protective effect appeared to originate from the interaction of Fab and IgG1 in small soluble oligomers, or through the rapid coalescence of pre-existing monomeric IgG1 nuclei into a dead-end aggregate, rather than through macromolecular crowding or diffusion-limitations.

## Introduction

Monoclonal antibodies (mAb) are a leading class of pharmaceutical products with over 80 approved products, and more than 570 products entering different stages of clinical trials as of 2019^1^. Among these products, most antibodies are formulated and administrated as individual therapeutic agents. However, recent clinical trials also report a number of combined administration routes showing promising results as new treatments^2–6^. The combination of nivolumab (Opdivo, anti-PD-1 antibody) and ipilimumab (Yvery, anti-CTLA-4 antibody), for example, inhibits two immuno-oncology checkpoints that shows encouraging response and survival rates in melanoma patients compared to the nivolumab single therapy^7,8^. There are a significant number of active clinical trials for combinations of other mAb products, typically involving 2–6 mAbs^9–11^, and in some cases up to 25^12^. As such clinical trials are beginning to reveal synergistic effects between multiple antibodies, this approach is becoming increasingly popular^3,13^. This increase, and also the extension beyond two-antibody therapies, will create a demand for coformulating these antibodies into a single product dosage form.

The mixing of antibodies raises potential new challenges for formulation in terms of degradation monitoring and control. Proteins are often presumed to become unstable when mixed, as the unfavourable interactions between proteins, coupled with an increased overall protein concentration can lead to earlier onset of aggregation. Minimisation of aggregation is a major challenge in almost every step of the manufacturing of mAb-based products, as this affects the final dosage of the pharmaceutically active product, loss of efficacy, and an increase in adverse immunogenicity upon administration^14–17^. Other than aggregation, fragmentation of a full-length antibody is also a key quality attribute that needs to be controlled during manufacturing, and also during the storage of the bulk and final dosage forms^18,19^. Peptide bonds at certain locations of mAbs are highly susceptible to proteolytic or acid-induced cleavage that leads to the formation of antibody fragments such as Fab, Fc or various heavy- and light-chain domains in solution. Thus, a formulation must provide the means to stabilize the combined molecules against these degradation pathways, during long-term storage^17,20–22^. As combination products are only recently emerging, there have been few studies into their stability in the mixture, with the aim of understanding how the multiple protein components interact and degrade during storage or in the final stages of drug product manufacturing^4,23^.

Here, we used a 144 kDa full-size monoclonal antibody (IgG1) and a 47 kDa antigen-binding fragment Fab as a model system to study the degradation of therapeutic proteins in coformulation. The IgG1 and the Fab were developed to bind to the A33 antigen, and so each have the same Fab sequence. This experimental design removed any complexity from having different CDRs, and also made the two proteins different in size to enable simpler separation by standard size-exclusion chromatography.

**Figure 1:**
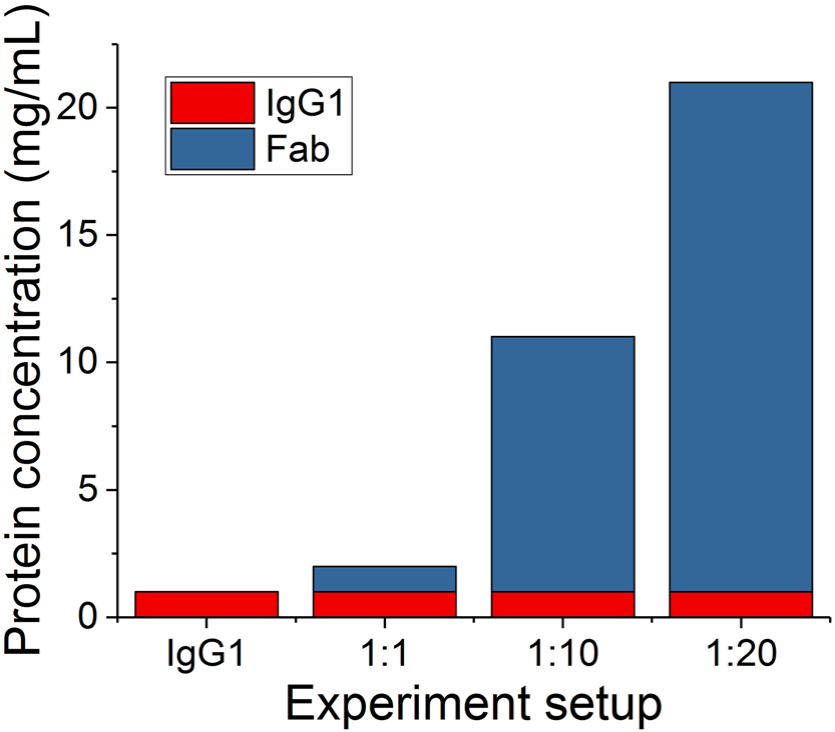
The experiment setup of IgG1-Fab coformulations. IgG1 and Fab was mixed at 1:1, 1:10 and 1:20 mass ratios with a fixed IgG1 concentration of 1 mg/mL. The IgG1 at 1 mg/mL was measured separately as a reference to its degradation in coformulation with Fab.

The IgG1 and Fab were mixed at 1:1, 1:10 and 1:20 (IgG:Fab) mass ratio, such that the concentration of the IgG1 was kept unchanged across all experiments, but that of Fab was increased to assess its impact on the stability of the IgG1 (Fig. 1). Moreover, IgG1 was also characterized separately as a reference system for the degradation in coformulations. The mixtures were stressed under moderately elevated temperature conditions to accelerate their degradation, while keeping the native structures intact. Thus, the aggregation would not be caused by interactions between hydrophobic core regions exposed in highly unfolded structures, but rather more closely reflects the mechanisms of degradation that would occur from native states and partially unfolded structures found under low-temperature storage^24–26^.

The degradation of the coformulated proteins was investigated using SEC-HPLC to assess the rates of monomer loss, as well as the appearance of fragments or soluble aggregates. The formation of larger particles was assessed by dynamic light scattering (DLS), and the formation of cross-beta sheets during aggregation was additionally assessed from the fluorescence of ThT dye-binding. These data showed that under the stress conditions, the IgG1 partially fragmented into a species with a very similar molecular weight to the Fab. It also showed aggregate formation at longer time-scales, though it was not determined whether these aggregates were formed from the entire IgG1 molecule, the Fab fragment produced earlier, or a mix of the two species.

The addition of bovine or human serum albumins (BSA or HSA), is known to stabilize many proteins and suppress their aggregation^27,28^. However, increasing the concentration of an antibody-based molecule is normally expected to increase aggregation or to reach the limits of solubility. For the coformulation of two related proteins, the effects of increasing one of the components has not been investigated previously^29^. Here we investigated increasing the concentration of only the Fab component of an IgG, in the presence of the same IgG molecule. If aggregation is driven primarily by self-interaction of the Fab region, then increasing the Fab concentration might be expected to accelerate the aggregation of the equivalent IgG. Surprisingly, we found that the aggregation of the IgG1 was gradually suppressed as the concentration of Fab was increased, suggesting that the Fab was in fact stabilizing. We investigated whether the Fab stabilized the IgG1 via a hard-shell macromolecular crowding effect. However, the macromolecular crowding agent Ficoll70 had no effect on stabilising IgG1 against aggregation, indicating that the Fab exerted its stabilising effect on aggregation kinetics through an alternative mechanism, such as a specific interaction between the Fab and the IgG1. Overall, our results show that coformulated therapeutic proteins could stabilize, at least in this case, rather than destabilize each other in protein mixtures.

## Results

The stability of the IgG1 and Fab coformulations were evaluated at pH 4.5, 100 mM ionic strength and 50 °C, which destabilized the IgG1 sufficiently for measurement of the degradation kinetics over an experimentally practical timescale. The structures of IgG1 and Fab in this buffer, showed no evidence of significant differences to those at pH 7.0, as probed by far-UV CD spectroscopy at 1 mg/mL of each protein independently (Supplementary Fig. S1).

### Thermal stability of the IgG1 and Fab coformulations

**Figure 2:**
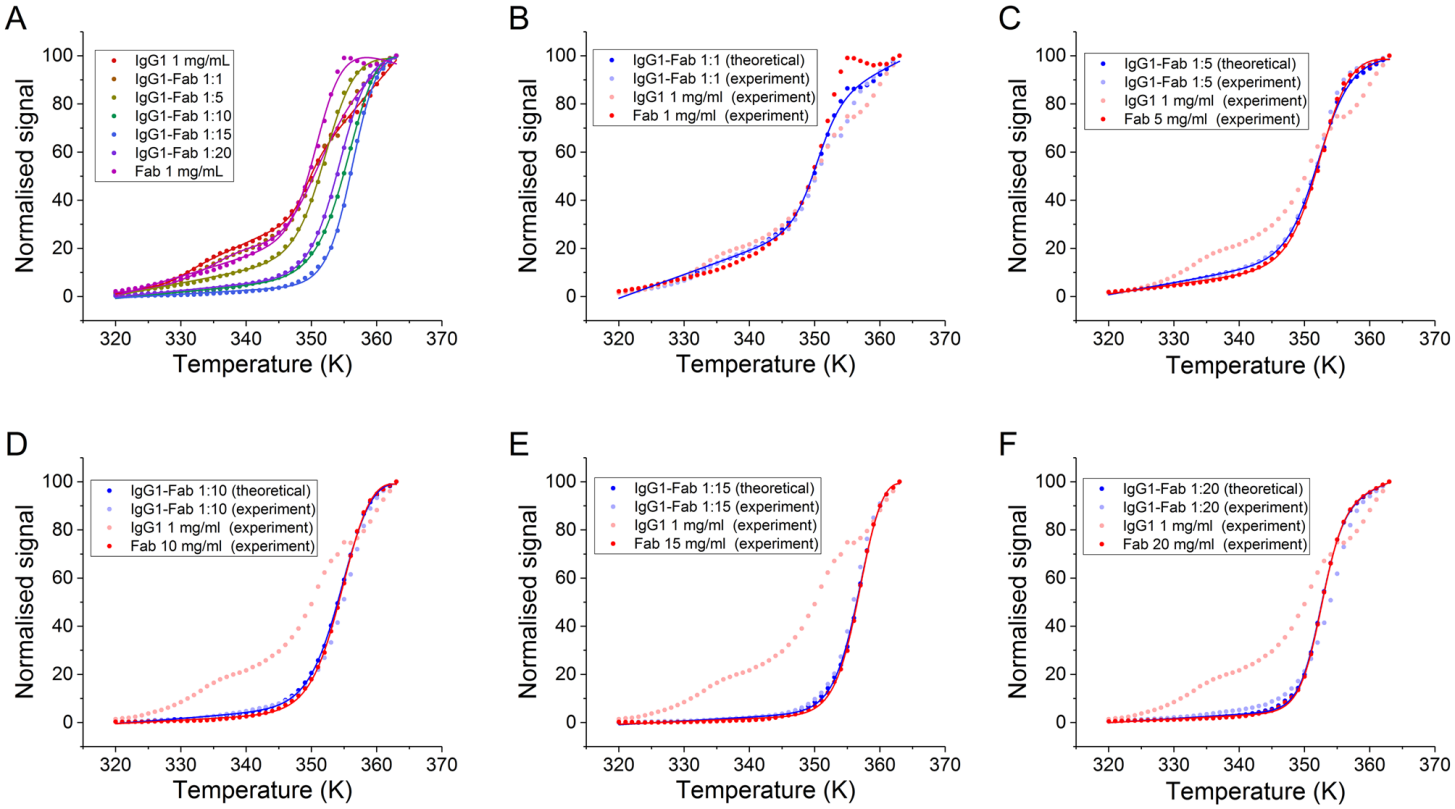
Thermal unfolding curve of the IgG1 and Fab. (**A**) The experimental data of IgG1 and Fab data of 1 mg/mL and coformulations of 1:1, 1:5, 1:10, 1:15 and 1:20 ratios are shown in rainbow colours, respectively. The temperature is displayed in *Kelvin* for the ease of fit. The curves are the least-squared fit of the data to a two- or three-state unfolding model. (**B–F**) Denaturation curves for the theoretical and experimental data for each coformulation, with the equal concentration of Fab as comparison.

Thermal denaturation curves were obtained for the IgG1, Fab and also IgG1-Fab mixtures, using intrinsic fluorescence as the structural probe to ascertain: (a) the appropriate temperature at which isothermal degradation kinetics should be carried out; and (b) the impact of coformulation on the conformational stability of the IgG1 and Fab, as reported by *T*_m,app_. The IgG1 at 1 mg/mL underwent two apparent transitions (Fig. 2). The curves were fitted to a three-state unfolding model and the resulting melting temperatures (*T*_m,app 1_ and *T*_m,app 2_) were 56 ± 3 °C and 75.7 ± 0.4 °C, respectively for each transition (Supplementary Table S1). By comparison, a single unfolding transition was observed for 1 mg/mL Fab, which fitted best to a two-state unfolding model to give *T*_m,app_ of 78.2 ± 0.2 °C. The *T*_m_ of the isolated Fab at 1 mg/mL, was therefore approximately 2 °C higher than for the same Fab fragment within the intact IgG1. This implies that the Fab fragment within the intact IgG1 could form interactions with either the other Fab- or Fc-region of the IgG1 that slightly destabilizes the internal Fab region during thermal denaturation, or makes it more susceptible to thermal aggregation. This is consistent with previous observations of the close packing of one Fab onto the Fc region in crystal structures and similar asymmetry in solution structures obtained by small-angle X-ray scattering (SAXS)^30^.

The concentration-dependence of the Fab *T*_m,app_ was then investigated from 1 to 20 mg/mL, both for Fab alone, and also in the presence of 1 mg/mL IgG1 (Fig. 2). Both of these protein components contributed to the fluorescence signals in the denaturation curves of IgG1-Fab coformulations, therefore the denaturation curve showed some of the early transition from IgG1 at 1:1 mixing. However, the first transition of the IgG1 was no longer observable at 1:5, 1:10, 1:15 and 1:20 (IgG:Fab), because the Fab concentration and corresponding signal became increasingly dominant. Therefore, the denaturation curve of the 1:1 coformulation was fitted using a three-state unfolding model, while the others were fitted using a two-state model. The *T*_m,app_ for the first transition at 1:1, was 67 ± 17 °C, with a high fitting error resulting from the transition being the poorly-defined minor component within the mixture (though consistent with the 56 ± 3 °C observed with IgG1 alone).

**Figure 3:**
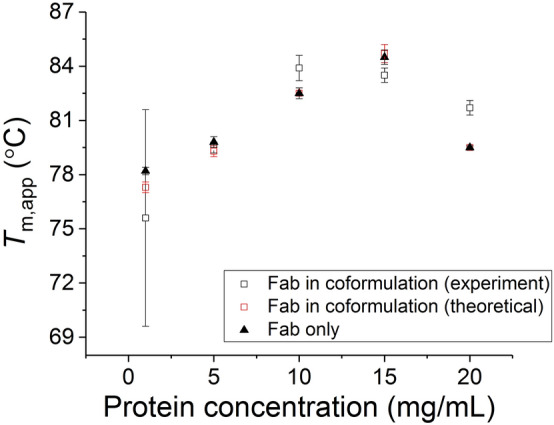
The *T*_m,app_ obtained from the experimental and theoretical data of Fab in coformulation is shown in black and red open squares (converted from Kelvin to Celsius unit), respectively. The *T*_m,app_ for Fab in isolation is shown as black filled triangle.

The stability of Fab alone increased with concentration (Fig. 3), reaching a maximum stability at 15 mg/mL. The second transition in the coformulations (*T*_m,app2_), which corresponded to the combined signals of the two Fab components, similarly increased from 76 °C in the 1:1 mixture, to 84 °C in the 1:10 and 1:15 mixtures, and then decreased to 82 °C at 1:20 (Fig. 3 and Supplementary Table S1). However, *T*_m,app2_ was increasingly derived from the dominant signal of the Fab added in coformulations. Therefore, it was necessary to first deconvolute the relative signals of the IgG1 and Fab, in order to isolate the impact of coformulation on the stability of the added Fab fragment, from that of the existing Fab region within the IgG1.

A comparison was made between the experimental melting transitions for the IgG1 and Fab coformulations at each mixing ratio, and theoretical curves also obtained for coformulations by simple addition of the transitions obtained from the two isolated proteins, weighted according to the mixing ratios (Fig. 2B–F). Fitting of the theoretical coformulation curves to thermal unfolding transitions gave the theoretical *T*_m,app2_ values shown in Supplementary Table S1, and plotted as a function of Fab concentration in Fig. 3. The theoretical *T*_m,app2_ of coformulations was consistent with the experimental *T*_m,app2_ values for isolated Fab, as expected given the dominance of the Fab signal in coformulations. However, it was notable that the theoretical curve did not match the experimental curve for the 1:20 coformulation, such that the experimental coformulations appeared to be more stable than expected from simple addition of the two components at mixing ratio higher than 1:15. This indicated that the stability of Fab in the coformulated systems had increased at the higher total protein concentration, relative to the isolated Fab at the same concentrations.

### Investigation of large molecular weight aggregates using DLS

Since the unfolding of the IgG1 started at a lower temperature (56.4 °C) than for Fab, an incubation temperature of 50 °C was selected for aggregation kinetics, to just maintain the native structure of IgG1 in the coformulation, while also accelerating the degradation. The size distribution of the isolated IgG1 reference system, and also in the IgG1-Fab coformulations, was investigated using DLS to monitor protein aggregation during incubation at 4 °C and 50 °C for comparison. Isolated IgG1 retained a hydrodynamic diameter of 12 nm, typical for IgG molecules at 1 mg/mL^31^ (Supplementary Fig. S2), throughout the incubation of 60 days under the non-degradation conditions of 4 °C. When incubated at 50 °C, the protein became more aggregated within 10 days, such that the main IgG1 peak broadened and shifted to 18 nm, while a small amount of higher molecular weight peaks also appeared, with mean diameters of 100–500 nm. This indicated aggregation to small oligomers, as well as to larger particles. The result was further supported by a ThT dye-binding assay for IgG1 which indicated the formation of aggregates containing cross-beta sheet structures over the first 10 days of incubation at 50 °C (Supplementary Fig. S3).

For coformulations incubated at 4 °C, after 40 days, a small amount of larger species appeared at 450 nm diameter, for the 1:1, 1:10 and 1:20 mixtures, with the highest accumulation in the 1:20 mixture (Supplementary Fig. S4). These were not observed in the isolated IgG1 at 4 °C. When the coformulated proteins were thermally stressed at 50 °C, this led to a much higher polydispersity, and a higher intensity from species in the 50–1000 nm range, than in the IgG1 reference at 50 °C. The aggregates of 100–1000 nm were also observed earlier for the coformulations, being apparent after only 5 days at 50 °C. However, given the increased contribution from Fab in the mixture, and the higher sensitivity of DLS towards larger particles, a direct kinetic comparison was not possible by DLS. Thus, we also looked at the rate of monomer loss by SEC-HPLC.

### Characterization of monomer retention using SEC-HPLC

**Figure 4:**
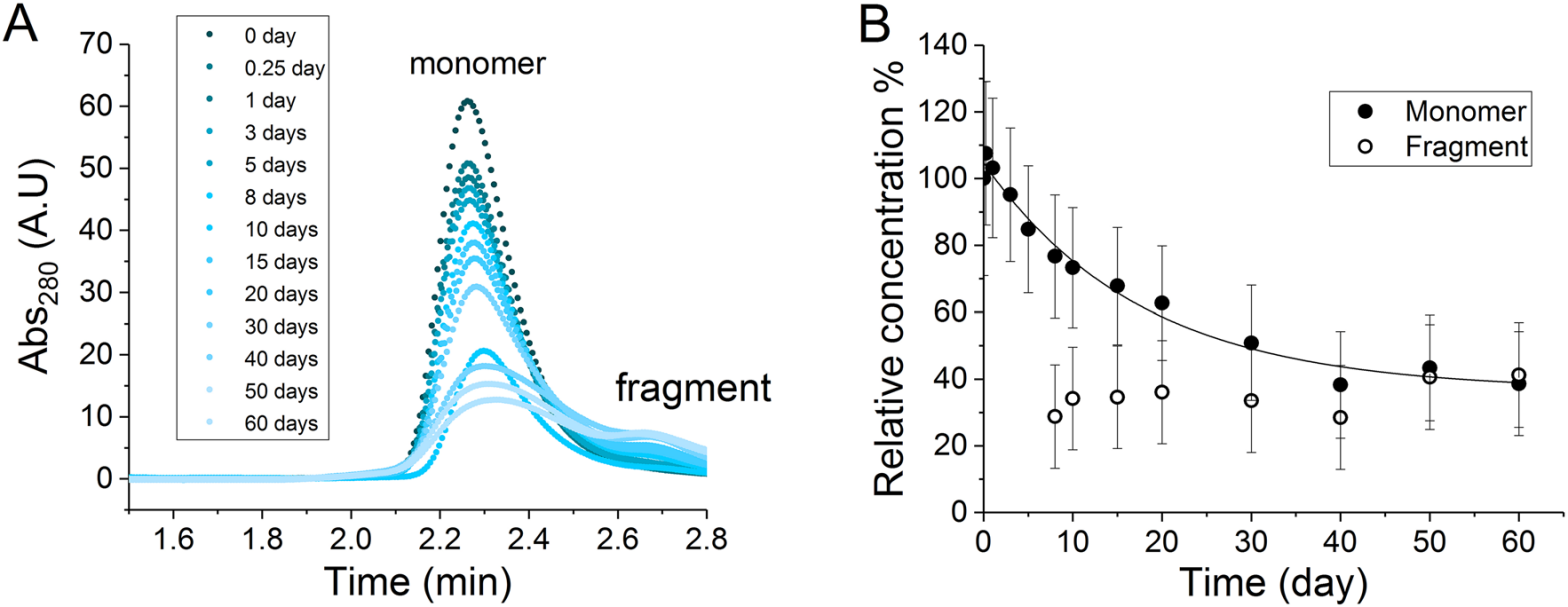
Degradation kinetics of IgG1 at 50 °C, pH 4.5. (**A**) Size exclusion chromatogram of IgG1 at 1 mg/mL from 0 to 60 days showing the emerging of a fragment species; (**B**) relative concentration of the IgG1 monomer and fragment species over experiment at 1 mg/mL. *A.U* arbitrary unit.

The degradation of the IgG1 was characterized using SEC-HPLC for 60 days at 50 °C, with measurements of identical samples incubated instead at 4 °C as a reference to remove any long-term instrument variability. Measurements of 1 mg/mL IgG1 degradation kinetics provided a baseline for comparison to subsequent coformulation experiments (Fig. 4). Moreover, to make a comparison with the 1:1 mixing of IgG1 and Fab (1 mg/mL of each), the degradation kinetics of an additional control sample of 2 mg/mL IgG1 was measured, to investigate whether any changes in the degradation kinetics of IgG1 were attributable simply to doubling of the total protein concentration (Supplementary Fig. S5).

The degree of IgG1 degradation was assessed via the loss of the monomer peak in SEC chromatograms. These also revealed the appearance of lower molecular weight species, likely to have resulted from partial fragmentation of the antibody (Fig. 4). The kinetics of monomer loss for both 1 and 2 mg/mL IgG1 was fitted to a simple first-order single-exponential equation to extract the rate constants, while the data of the first 20% monomer loss was also fitted to a linear equation to calculate the initial rate (Table 1). A total of approximately 50–60% monomer was degraded for both 1 and 2 mg/mL IgG1 over the period of experiment.

**Table 1:** Rate constant and initial rate extracted from the degradation kinetics of single- and co-formulations at 50 °C, pH 4.5.

	*k* (day^−1^)	Initial rate *v* (% day^-1^)	Monomer % at Day 60
IgG1 at 1 mg/mL	0.05 ± 0.01	3.0 ± 1.0	39 ± 16%
IgG1 at 2 mg/mL	0.04 ± 0.01	2.1 ± 0.5	40 ± 6%
IgG1 in 1:1 coformulation	0.04 ± 0.01	1.2 ± 0.2	68 ± 6%
Fab in 1:1 coformulation	N.D	− 0.05 ± 0.02	106.2 ± 0.7%
IgG1 in 1:10 coformulation	N.D	0.61 ± 0.05	68 ± 1%
Fab in 1:10 coformulation	N.D	0.07 ± 0.04	99 ± 1%
IgG1 in 1:20 coformulation	N.D	0.08 ± 0.06	98 ± 1%
Fab in 1:20 coformulation	N.D	0.07 ± 0.03	99.2 ± 0.3%
IgG1 in 20 mg/mL Ficoll70	0.04 ± 0.01	2.3 ± 0.6	52 ± 6%
IgG1 in 100 mg/mL Ficoll70	0.07 ± 0.05	7.0 ± 1.0	39 ± 3%

*N.D.* not determined.

**Figure 5:**
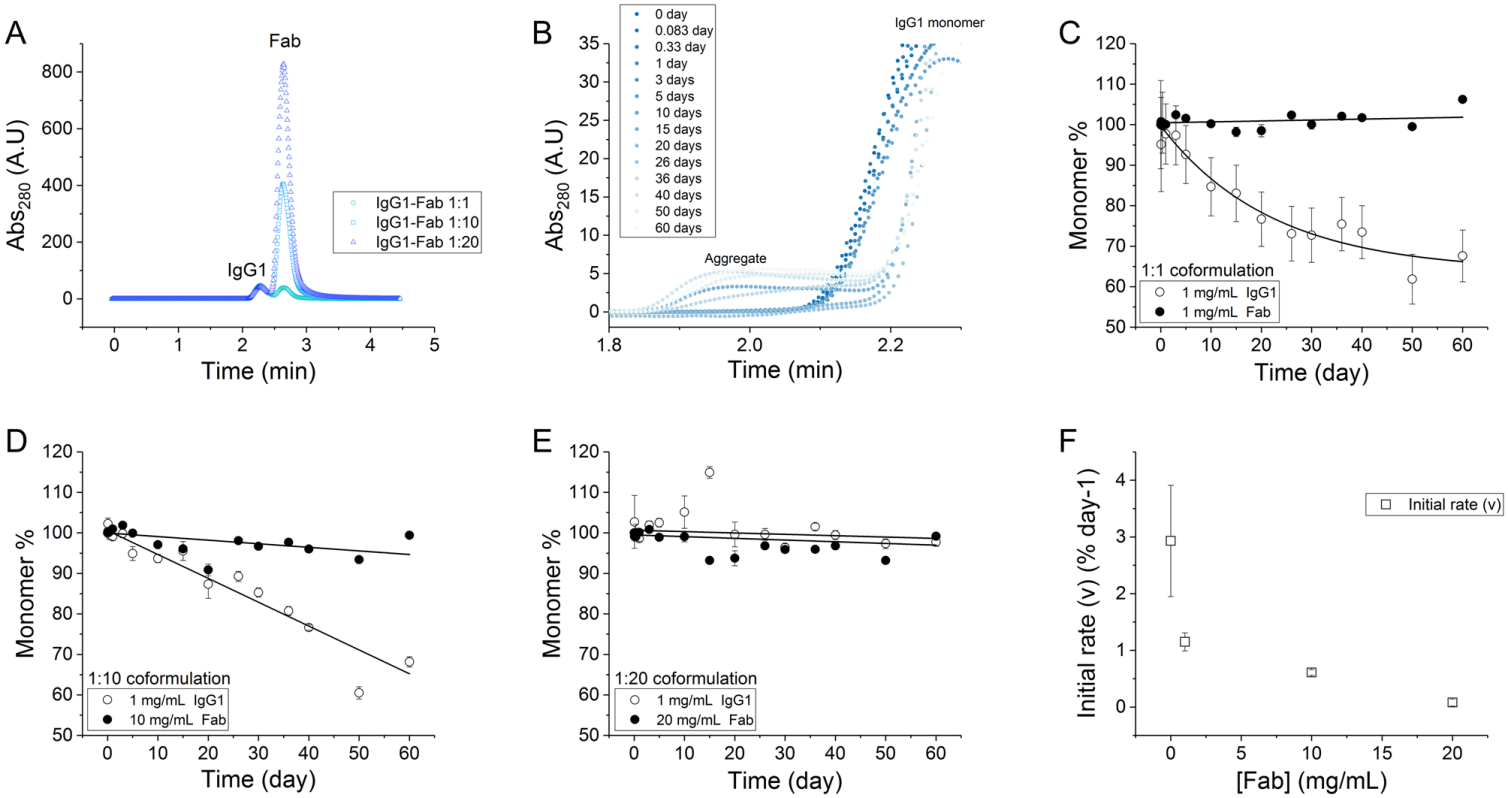
Degradation kinetics of IgG1-Fab coformulations at 50 °C, pH 4.5. (**A**) SEC profile of IgG1-Fab coformulations; (**B**) aggregate peak for IgG1-Fab coformulations (1:1 mixing); (**C–E**) Peak area over time of IgG1 and Fab monomer in coformulations. The monomer retention (monomer %) is obtained by normalizing the peak areas of monomer over the experimental time scale. The continuous lines show the best fit of the data to exponential or linear equations. (**F**) The change of initial rate of monomer loss of IgG1 over concentration of Fab. *A.U* arbitrary unit.

The degradation kinetics of the IgG1 and Fab coformulations are shown in Fig. 5. The IgG1 in the 1:1 coformulation followed a similar degradation profile to those of the controls of 1 mg/mL and 2 mg/mL IgG1 alone (Fig. 5C). The kinetics again fitted well to a simple first-order single-exponential equation, while the first 20% of monomer loss was also fitted to a linear equation to extract the initial rate (Table 1). IgG1 in the 1:1 coformulation had a similar rate constant for monomer loss at 50 °C, as those of the 1–2 mg/mL IgG1 control measurements. However, the initial rate of IgG1 in the 1:1 coformulation was around 30–50% of those for the IgG1 controls. The fragmentation of IgG1 in the coformulation could not be readily identified given that the added Fab had the same elution volume by SEC. The presence of this fragment might be inferred from the slight increase of the Fab peak over time in the 1:1 coformulation. However, this increase was not statistically robust, and so not possible to quantitatively correlate to any specific degradation in the mixture. In total, approximately 30–40% of the monomer was degraded after 60 days at 50 °C, compared to 60% for the IgG1 control measurements. Thus, despite their similar rate constants, the initial rate and extent of IgG1 degradation was suppressed by addition of the Fab into the 1:1 coformulation.

The degradation profile of IgG1 at the 1:10 mixing ratio was very different from those at 1:1 and in the absence of Fab, such that the IgG1 monomer loss no longer followed exponential kinetics (Fig. 5D). Instead there was a linear decrease in IgG1 monomer, and so it was not possible to extract the kinetic parameters from simple exponential kinetic models, as obtained in the previous measurements. However, the total monomer loss after 60 days, of approximately 30% at 1:10, was similar to that at 1:1. Likewise, the change in Fab concentration was very small at 1:10 over the period of the experiment.

For 1:20 mixing, both the IgG1 and Fab showed only a very small loss of monomer species over the 60-day period (Fig. 5E). The data of IgG1 and Fab were fitted to a simple linear equation to extract the initial rates, and were each found to be less than 0.1% per day (Table 1). Thus, a remarkable observation was made that with increasing concentration of Fab, the degradation of IgG1 was diminished until it was effectively inhibited at the 1:20 mixing ratio (Fig. 5F).

### Investigation of macromolecular crowding effects using Ficoll70

**Figure 6:**
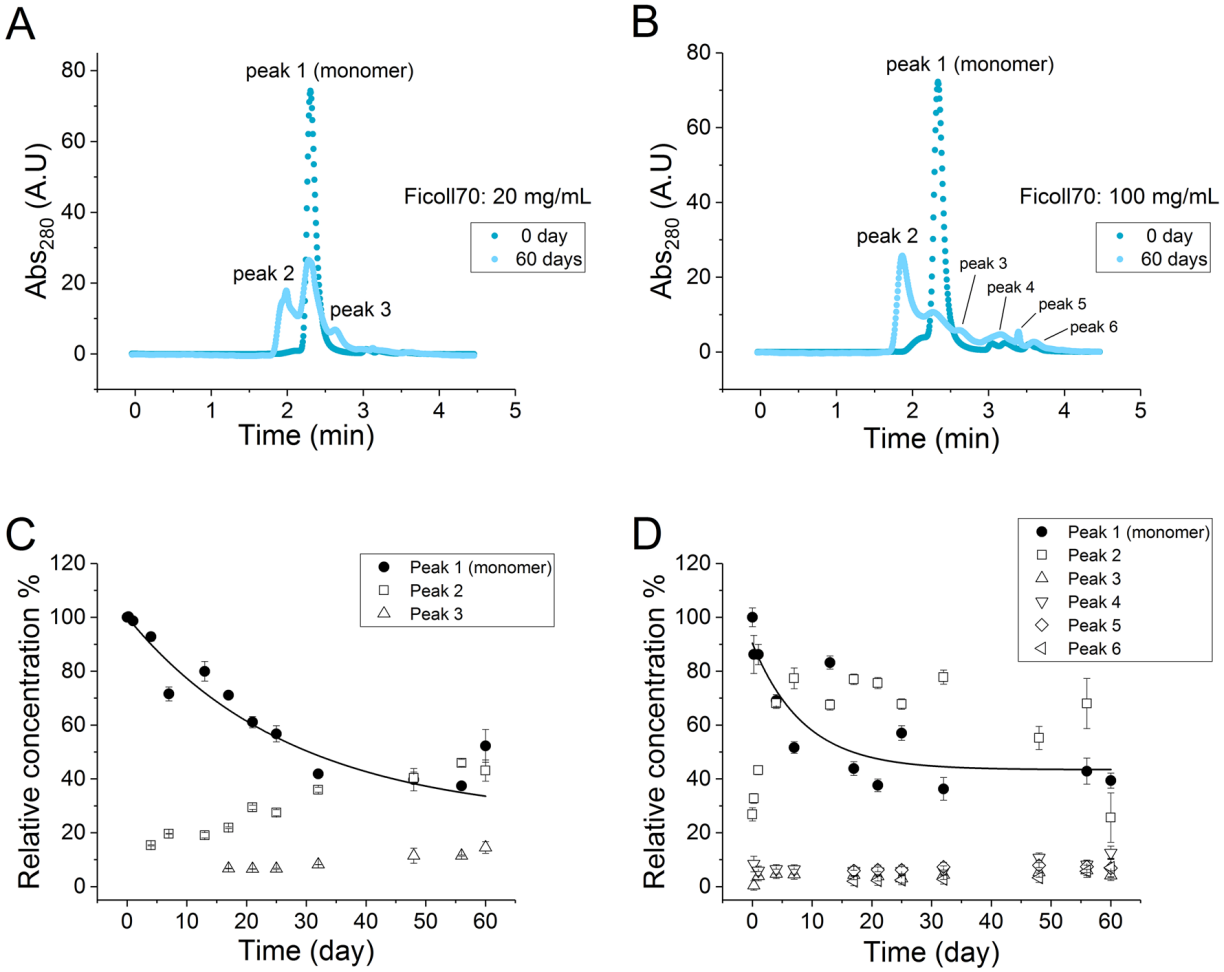
Degradation kinetics of IgG1 in Ficoll70. (**A**,**B**) SEC profile of 1 mg/mL IgG1 mixed with 20 mg/mL and 100 mg/mL Ficoll70 shown at day 0 and day 60; (**C**,**D**) Change in specific peak area over time for the monomer and degradation species over 60 days period. *A.U* arbitrary unit.

We investigated whether the cause of the IgG1 stabilization was a molecular crowding effect due to the addition of the Fab, by replacing the Fab with the sucrose polymer Ficoll70 which is a well-known crowding agent^32–34^. The final concentrations of Ficoll70 were 20 and 100 mg/mL to explore different levels of crowding. The SEC chromatograms of IgG1 in the presence of Ficoll70 were plotted for the native and heat-stressed samples (Fig. 6). In all cases, the changes in the relative concentration of the monomer and aggregate were fitted with a first-order single-exponential equation to extract the kinetic rate constants and the initial rates obtained by fitting the first 20% signal change to a linear equation (Table 1).

The IgG1 in 20 mg/mL Ficoll70 gave a major peak and a minor higher molecular-weight peak at day 0. After 60 days of stress, considerable aggregation and fragmentation occurred to give higher and lower molecular weight peaks. The peak distribution at the end of the experiment was similar to that for the IgG1 control system and the coformulations, but the higher molecular weight aggregate peak was much stronger in the presence of 20 mg/mL Ficoll70. In the presence of 100 mg/mL Ficoll70, IgG1 produced a more complex degradation result with at least 6 peaks observed by SEC at the end of the experiment (Fig. 6). In addition to the aggregate peak which is observed in other measurements, IgG1 in 100 mg/mL Ficoll70 led to a few additional peaks.

After 60 days, the IgG1 monomer remaining was 40% for both 20 mg/mL and 100 mg/mL Ficoll70, and therefore comparable to the 50–60% monomer loss for 1 mg/mL IgG1 alone. The rate constants of monomer loss for IgG1 in the absence and presence of Ficoll70 were also similar (0.05–0.06 day^−1^), while the initial rate at 100 mg/mL Ficoll70 was slightly higher (accounting for the errors) than in the other two conditions. Together, these data suggested that Ficoll70 at this concentration had no significant impact on the aggregation rate of IgG1.

## Discussion

Few studies have investigated the individual stability and degradation of therapeutic protein components in coformulations^4,5,35^. Therefore, there is no current consensus or strategy for guiding the development of stable coformulated products. It is currently difficult to predict the degradation pathways and kinetics of individual components in a protein mixture. While some proteins, notably the albumins, are already used as formulating agents that can stabilize other proteins^27^, combinations of antibody-based proteins might easily be presumed to become more aggregation prone than the isolated antibodies, simply as a result of increasing the total IgG concentration. However, many other factors may need to be considered, such as macromolecular crowding or volume exclusion effects, and the protection from aggregation by increased interactions with non-identical proteins. In this specific IgG1-Fab coformulation study, we have shown that two therapeutic proteins can provide an additional stability benefit compared to formulations of the single antibody.

A moderate elevated temperature of 50 °C, at pH 4.5, was used to stress the formulations, as the fraction of unfolded protein was therefore kept below 1.5% for the Fc region of the IgG1, and below 0.04% for the Fab region (Supplementary Fig. S6). This ensured that degradation kinetics related to interactions between native proteins, as they would be at lower temperature storage conditions, yet enabled kinetic studies on a practical timescale. The structural integrity of IgG1 and Fab was confirmed at pH 4.5 using far-UV CD, showing minimal structural differences to those at pH 7.0. Under the conditions of 50 °C at pH 4.5, the kinetic measurements spanned up to 60 days to generate a significant monomer loss, compared to the much shorter timescales of several hours, used in studies with more *T*_m_-proximal aggregation temperatures, and hence also dominated by aggregation from globally-unfolded protein^24^. Fragmentation was a major form of degradant for IgG1 under the stressed condition, and could be detected earlier than the minor aggregation species. This suggested that the degradation pathway of IgG1 was biased towards fragmentation under this condition, which may result from the acidic environment that leads to hydrolysis of the hinge region that connects Fab to Fc. However, the specific involvement of the fragment in the aggregation of the intact monomer, was not investigated further due to its analytical complexity.

The aggregate population induced in IgG1 was polydisperse. The largest size was approximately 1000 nm, consistent with large oligomeric intermediates in previous reports for IgG molecules^36^. Larger aggregate particles were also visible by eye at the end of incubations, and such large aggregates would have been removed by the centrifugation prior to SEC analysis. For IgG1-Fab coformulations, the size distribution showed much higher polydispersity than for the isolated 1–2 mg/mL IgG1 references. This indicated an interaction between the IgG1 and Fab, at some level, such that a wider distribution of aggregated species of different sizes was formed. The initial formation of aggregates (100–1000 nm) was slightly faster in the coformulations as measured by DLS. This was an interesting result when compared to the slowed rate of monomer loss as measured by SEC. Certainly, DLS is highly sensitive and biased towards larger particles, and so the quantity of the small aggregates detected by DLS would be relatively low. However, it suggests that the coformulated material produces an initial population of soluble aggregates more rapidly, but that these potentially contribute to inhibition of further monomer loss, perhaps by rapidly removing pre-existing nucleation sites from free solution.

SEC was readily able to resolve and quantitate the IgG1 and Fab over time. The higher monomer retention for IgG1 in the 1:1 coformulation indicated that the degradation of IgG1 was slowed by the presence of Fab. This was further enhanced in the 1:10 and 1:20 coformulations, such that the degradation kinetics of IgG1 in the 1:20 coformulation were hardly observable within the experimental time-scale. A previous study showed that the Fab at pH 3.5, formed minor levels of aggregates that eluted prior to the monomer^24^. However, the elution time of these aggregates was still later than that of IgG1, and so the slowed monomer loss in the presence of Fab was not simply due to the co-elution of IgG1 monomer and Fab aggregate peaks.

The stability of therapeutic proteins in combination is rarely reported for pharmaceutical products, but there are many examples for which the coformulation with protein-based excipients such as albumin, increased the stability of the pharmaceutical ingredient^37–39^. Given that Fab is already known to be relatively stable under the stress conditions, it is possible that the Fab acted in a similar way to that often observed with albumins. However, a direct comparison of the IgG1 coformulated with recombinant human serum albumin was not possible as the albumin failed to tolerate the stress conditions used in this study (data not shown).

The conformational stability (melting temperature) of the Fab fragment increased at higher Fab concentrations both for Fab alone, and in the coformulation with IgG1. Meanwhile, the melting temperature of the Fab within 1 mg/mL IgG1 was slightly lower than for free Fab, suggesting the presence of destabilising intramolecular interactions either between the two Fab regions, or between Fab and the Fc. Stabilisation of the IgG1 degradation kinetics may therefore, also have derived from the increased conformational stability of its Fab region, as extra Fab was added. It was not possible to track the conformational stability of the Fc region in the presence of excess Fab, and so it remains also possible that the excess Fab had similarly stabilized the critical Fc region which unfolded at the lower temperature. In future, the specific mechanism and relative roles of the Fc and Fab could be investigated through varying the IgG1, Fab and Fc components independently.

The decrease in degradation kinetics for IgG1 in the presence of increased Fab was postulated to be achievable via the following mechanisms:

1. macromolecular crowding by Fab which stabilizes all protein components in the mixture by decreasing the available volume for protein unfolding or native-state fluctuations;
2. decreased diffusional collision between IgG1 molecules due to viscosity effects, or molecular screening in the presence of Fab;
3. direct interactions between the Fab and IgG1 that stabilized the IgG1 against partial unfolding or interaction with other IgG1 molecules.
4. added Fab induces the rapid formation of dead-end aggregates that mopped up all pre-existing nucleation-site containing IgG1, and inhibited further monomer loss.

To test the first potential mechanism, a widely used non-reducing macromolecular crowding agent Ficoll70 was added at 20 mg/mL and 100 mg/mL to create different degree of crowding environment for IgG1. However, 20 mg/mL Ficoll70 had no impact on the IgG1 degradation rate, whereas 100 mg/mL Ficoll70 doubled the degradation rate, which indicated that the crowding environment actually promoted the degradation of IgG1. The addition of Ficoll70 will have effectively concentrated the protein, which could promote diffusion-limited collisions, or change the solvation properties of the protein. However, the addition of Fab would be expected to do the same. Moreover, the IgG1 produced an increased range of fragments in the presence of the high concentration of Ficoll70, which suggested that the protein was further fragmented into individual domains of the heavy and light chain. This was an unexpected result as a macromolecular crowding environment is often expected to favour a more compact protein conformation that would more likely prevent the partial unfolding and conformational fluctuations within the native protein ensemble^34,40,41^. However, other evidences have shown that a crowding environment could lead to destabilization of the native state of the protein by creating non-specific interactions between the protein and crowder molecule or by increasing protein–protein interactions in the solvent^42,43^. As a result, it appears that the Fab did not stabilize the IgG1 via simple macromolecular crowding, although it remains possible that Fab does still exert macromolecular crowding through a different mechanism to that expected from Ficoll.

Given that the Fc region of the IgG1 unfolded at a lower temperature than the Fab, then the Fc region is likely to play a major role in the aggregation of IgG1 under these conditions. As a result, Ficoll70 could crowd the IgG1 molecules such that self-interactions between Fc regions are promoted, which drives the degradation of the molecule. However, the same would be expected from the addition of the Fab, and so it seems more likely instead, that the Fab interacts directly with the Fc region of IgG1 to protect it from self-interaction between Fc regions, in a way that Ficoll70 does not. Thus mechanism 3 (above), will need to be investigated by other biophysical approaches, along with the related mechanism 4 that was suggested from the DLS results.

It will be of interest for pharmaceutical industries to understand whether the stabilization in coformulated therapeutic proteins can be regulated by changing or engineering the mixing components towards certain properties that favours coformulation. For now it is promising that the coformulation of an IgG1 with an equivalent Fab fragment leads to a more stable formulation of the IgG1, as this presents the potential for rapid development of treatments, for example using multi-sized proteins with different pharmacokinetic properties, but with the same target in vivo, to be able to tune the overall range of dosing flexibility.

## Materials and methods

### Materials

The IgG1 protein, and the *E. coli* strain W3110 containing pTTOD A33 IGS2 for Fab expression were obtained from UCB (Slough, UK). A C226S variant of Fab was used as earlier described to reduce dimerization^24^. All reagents including buffers, inorganic salts and Ficoll 70 (72146-89-5) are analytical grade and purchased from Sigma-Aldrich (Poole, UK).

### Methods

#### Protein production and purification

A pilot-scale production of the Fab was carried out in a 30 L fermenter (BIOSTAT Cplus, Sartorius, Goettingen, Germany) and purified using sequential AKTA-based liquid chromatography as described elsewhere^24^. The purified protein was dialysed into ultrapure water at 4 °C for overnight using Dialysis Cassettes, 10 K MWCO (Thermo Scientific, 66811) and concentrated to 36.75 mg/mL prior to dilution into desired concentrations and buffers.

IgG1 was purified using gel filtration (HiLoad Superdex 75, GE Healthcare) to remove a minor large molecular weight species and the monomer peak was pooled and dialysed into ultrapure water. The purified protein was concentrated to 10 or 20 mg/mL prior to dilution into desired concentrations and buffers.

### Sample preparation

The measurements were carried out for the IgG1 : Fab coformulations at three mass-mixing ratios: 1:1, 1:10 and 1:20. The concentration of IgG1 was 1 mg/mL in all coformulations and those of Fab were 1, 10 and 20 mg/mL, respectively. IgG1 was also measured individually at 1 mg/mL and 2 mg/mL as references. The stressing buffer used in measurement was 20 mM sodium acetate (pH 4.5) and the final ionic strength was adjusted to 100 mM by adding NaCl.

### Thermal melting

The apparent thermal stability of the IgG1, Fab and IgG1-Fab coformulations were each measured using a UNit system (Unchained Labs, Pleasanton, US). The protein was step-heated from 20 to 95 °C at 1 °C/min and with 30 s equilibration at each temperature. The intrinsic fluorescence spectrum at each temperature was recorded in triplicate. The BCM (Barycentric Mean) of each spectrum was calculated by the instrument software and plotted against temperature. In all cases it is assumed that rapid equilibrium unfolding is convoluted with irreversible aggregation from the unfolded protein during thermal ramping, and hence *T*_m_ values are presented as apparent, *T*_*m,app*_. However, the measured *T*_*m,app*_ values are reproducible for a given thermal ramp rate, and provide a measure of the conformational stability that allows comparison between formulations.

Depending on whether or not an intermediate state was populated in the denaturation curve, the data was fitted using either a two-state (Eq. 1) or three-state (Eq. 2) unfolding model to extract the apparent midpoint of unfolding transitions (*T*_*m,app*_):

1 $$ I_{T} = \frac{{\left( {I_{N} + aT} \right) + \left( {I_{D} + bT} \right)K}}{1 + K} $$

with an equilibrium constant for the transition between the native and denatured state,

$$ K = \exp \left[ {\frac{{\Delta H_{vh} }}{R} \left( {\frac{1}{{T_{m,app} }} - \frac{1}{T}} \right)} \right]. $$

In these equations, *T* is the experimental temperature; *T*_*m,app*_ is the temperature at which the protein is half denatured; *I*_*T*_, *I*_*N*_ and *I*_*D*_ are the spectroscopic signals of the protein at each given temperature, at the native and at the fully denatured state, respectively. *a* and *b* are the baseline slopes of the native and denatured region of the curve. *ΔH*_*vh*_ is the van’t Hoff enthalpy and *R* is the gas constant. All temperature terms in this equation are absolute temperatures in *Kelvin*.

2 $$ I_{T} = \frac{{\left( {I_{N} + aT} \right) + \left( {I_{I} + bT} \right)K_{1} + \left( {I_{D} + cT} \right)K_{1} K_{2} }}{{1 + K_{1} + K_{1} K_{2} }}. $$

*K*_1_ is the equilibrium constant for the transition between the native and intermediate state and *K*_2_ is the equilibrium constant between the intermediate to denatured states, where.

$$K_{1} = \exp \left[ {\frac{{\Delta H_{vh1} }}{R} \left( {\frac{1}{{T_{m,app 1} }} - \frac{1}{T}} \right)} \right]\,{\text{and}}\,K_{2} = \exp \left[ {\frac{{\Delta H_{vh2} }}{R} \left( {\frac{1}{{T_{m,app 2} }} - \frac{1}{T}} \right)} \right].$$

In these equations, *T* is the experimental temperature; *T*_*m,app 1*_ and *T*_*m,app 2*_ are the temperatures at which the protein is half denatured for the transition of native to intermediate and intermediate to denatured states, respectively; *I*_*T*_, *I*_*N*_* I*_*I*_ and *I*_*D*_ are the spectroscopic signals of the protein at each given temperature, at the native, intermediate and fully denatured state, respectively. *a, b* and *c* are the baseline slopes of the native, intermediate and denatured region of the curve. *ΔH*_*vn1*_* and ΔH*_*vn2*_ are the van’t Hoff enthalpy for both transitions and *R* is the molar gas constant. All temperature terms in this equation are absolute temperatures in *Kelvin*.

The apparent mole fraction of the unfolded species was given by:

3 $$ f_{app} = \frac{{\exp \left[ {\frac{{\Delta H_{vh} }}{R}\left( {\frac{1}{{T_{m} }} - \frac{1}{T}} \right) } \right]}}{{1 + \exp \left[ {\frac{{\Delta H_{vh} }}{R}\left( {\frac{1}{{T_{m} }} - \frac{1}{T}} \right) } \right]}}, $$

where *f*_app_ is the apparent mole fraction of the unfolded species at temperature T.

### Dynamic light scattering

The dynamic light scattering (DLS) of the IgG1 and Fab samples was measured using a Zetasizer Nano ZSP system (Malvern, UK) at 13° and 173° angle using a reference viscosity of the stressing buffer. Each scattering curve was obtained from 15 repeated scans. The samples were measured in triplicate at 20 °C.

### ThT assay

ThioflavinT (ThT) fluorescence in the presence of IgG1 was measured during isothermal incubation with a FLUOstar Omega microplate reader (BMG Labtech, UK). ThT was mixed with the protein at a final concentration of 50 μM. The samples were excited at 445 nm and the fluorescence signal recorded at 490 nm. All kinetic measurements were carried out in a final volume of 100 μL in triplicates with a final IgG1 concentration of 1 mg/mL.

### Far-UV circular dichroism

The IgG1 and Fab were diluted into the stressing buffer (sodium acetate, pH 4.5, 100 mM ionic strength) and a control 20 mM phosphate (pH 7.0) buffer, individually to allow for equilibration over 24 h at 25 °C. The CD spectra were collected on an AVIV CD spectropolarimeter (Lakewood, US) thermostatted at 20 °C. A 1.0 mm pathlength cuvette was used throughout and the spectra were scanned at 1 nm per second for the range of 200–260 nm. Each spectrum was averaged over five repeated measurements.

### Aggregation kinetic measurement using SEC-HPLC

For measurement of IgG1-Fab coformulations and the IgG1 control, proteins were incubated at 4 °C and 50 °C, respectively, in tight-lid Eppendorf tubes to minimize evaporation for up to 60 days. The results of 4 °C samples were used as a control reference for 50 °C stressed samples. Aliquots were taken at each timepoint, and their aggregation quenched by centrifugation at 11,000*g* for 45 min at 4 °C. The soluble portion of the sample was then transferred to a 96-well microplate or glass vial, held at 4 °C, prior to analysis on a Zorbax-GF250 size-exclusion chromatography (SEC) column at 1 mL/min on an HPLC instrument (1200 series, Agilent, UK), using 200 mM sodium phosphate pH 7.0 as the mobile phase with the column at room temperature. Elution profiles at 280 nm were averaged over at least three repeated measurements. Peaks were fitted to a single or double Extreme equation (Eq. 4) in OriginPro 2016 (OriginLab, UK) to best account for the tailing of the chromatogram:

4 $$ y = y_{0} + A\exp \left( { - \exp \left( { - z} \right) - z + 1} \right), $$

where $$z = \frac{{\left( {x - xc} \right)}}{w}$$.

In these equations, *y* is the absorbance, *y*_0_ is the offset of the chromatogram, *x* is the elution time, *xc* is the center of the peak, *w* is the width, *A* is the amplitude. Then the area under the peak was obtained by integration.

The change in the relative concentration of monomer and other degradation species was calculated by subtracting the monomer peak area measured for the 4 °C samples, by from those of the 50 °C stressed samples, and normalized over the peak area at day 0:

5 $$ normalised\, signal = \frac{{A_{0} - \left( {A_{{4\,^\circ {\text{C}}}} - A_{{50\,^\circ {\text{C}}}} } \right)}}{{A_{0} }} \times 100, $$

where $A_{0}$ is the peak area at time zero, $A_{{4\,^\circ {\text{C}}}}$ and $A_{{50\,^\circ {\text{C}}}}$ are the peak area of 4 °C and 50 °C at each given incubation time.

## Conclusion

Degradation kinetics of the mixtures of two therapeutic proteins, IgG1 and Fab, were investigated under mildly destabilizing conditions. The degradation of the IgG1 was significantly inhibited by the excess of Fab in the mixture, even though the initial population of a small amount of mid-sized aggregates was more rapid. The mechanism appears not to be due to macromolecular crowding or diffusion limitation effects at the higher concentration of Fab, as probed by adding Ficoll70. Rather, it appears to be linked to specific protective interactions between, Fab and IgG1, either through forming small soluble oligomers (mechanism 3), or through the rapid coalescence of all available monomeric IgG1 nuclei into an initial dead-end aggregate that slows any further aggregation. This role of the protection by Fab is not reported in any earlier studies and could shed some light on the future development of antibody combination products. Whether the inhibition of degradation is specific to this protein combination, or extends more generally to other combinations, will be interesting to investigate in future, as it could provide a new rationale for designing stable coformulated antibody products.

## Supplementary information

Supplementary Information.
