# Supplementary material for: Stability enhancement in a mAb and Fab coformulation
Source: Sci Rep. 2020 Dec 3;10:21129. doi: 10.1038/s41598-020-77989-w (PMC7713237; doi:10.1038/s41598-020-77989-w)
Supplement: Supplementary file 1 — Supplementary Information. [file 41598_2020_77989_MOESM1_ESM.pdf]

Supplementary information

Stability enhancement in a mAb:Fab protein coformulation

Hongyu Zhang[1,2], Paul A. Dalby[1,*]

1 Department of Biochemical Engineering, UCL, London, UK, WC1E 6BT

2 EPSRC Future Targeted Healthcare Manufacturing Hub, UCL, London, UK, WC1E 6BT

*Correspondence to: p.dalby@ucl.ac.uk

IgG1 or Fab in isolation	IgG1 (1 mg/mL)	Fab (1 mg/mL)	Fab (5 mg/mL)	Fab (10 mg/mL)	Fab (15 mg/mL)	Fab (20 mg/mL)
$T_{m,app\ 1}$ (°C)	56 ± 3	N.A.	N.A.	N.A.	N.A.	N.A.
$T_{m,app\ 2}$ (°C)	75.7 ± 0.4	78.2 ± 0.2	79.8 ± 0.3	82.5 ± 0.3	84.5 ± 0.4	79.5 ± 0.1
Fab in coformulation (experimental)		1:1	1:5	1:10	1:15	1:20
$T_{m,app\ 1}$ (°C)		67.0 ± 17.4	N.D.	N.D.	N.D.	N.D.
$T_{m,app\ 2}$ (°C)		75.6 ± 6	79.5 ± 0.3	83.9 ± 0.7	83.5 ± 0.4	81.7 ± 0.4
Fab in coformulation (Theoretical)		1:1	1:5	1:10	1:15	1:20
$T_{m,app\ 1}$ (°C)		N.A.	N.A.	N.A.	N.A.	N.A.
$T_{m,app\ 2}$ (°C)		77.3 ± 0.3	79.3 ± 0.3	82.5 ± 0.3	84.7 ± 0.5	79.5 ± 0.1

TABLE S1: Thermodynamic parameters of the IgG1 and Fab in individual or in coformulation measurements. The experimental $T_{m,app}$ was obtained from either three- or two-state unfolding model. The theoretical value was calculated by fitting the denaturation curves which were obtained by simple addition of the curves of each mixing component by their mixing ratio. N.A.: not applicable. N.D.: not determined

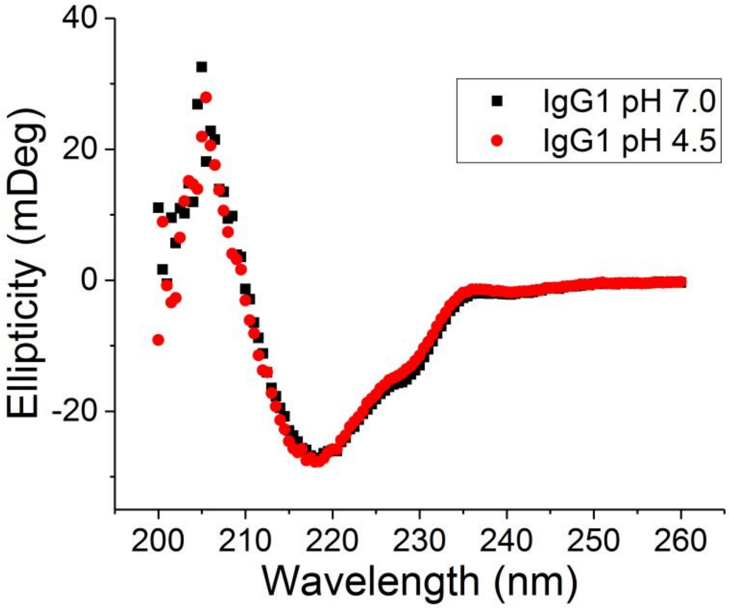

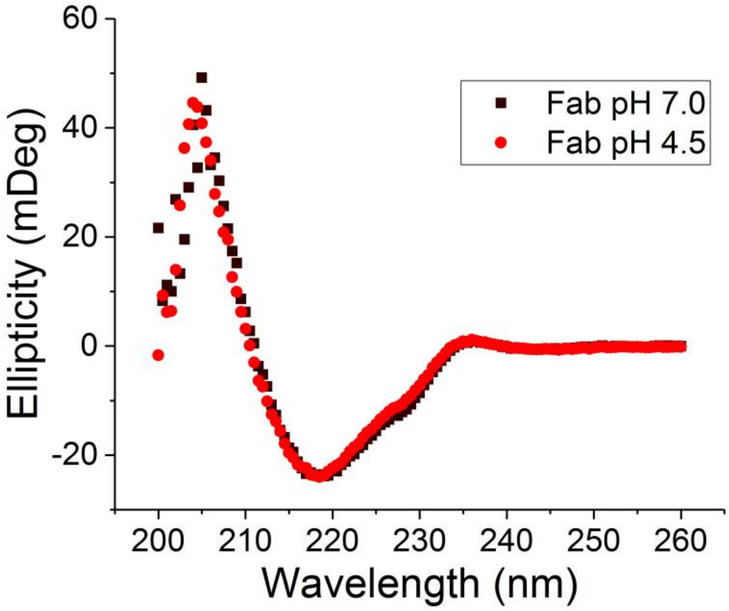

FIGURE S1: Far-UV circular dichroism spectra of the IgG1 and Fab each at 1 mg/mL after 24 hours of equilibration at 20 °C. The red filled circles represent the spectra of proteins incubated in the stress buffer of 20 mM sodium acetate, pH 4.5, 100 mM ionic strength. The black filled squares represent the spectra of proteins incubated in native buffer (20mM phosphate, pH 7.0).

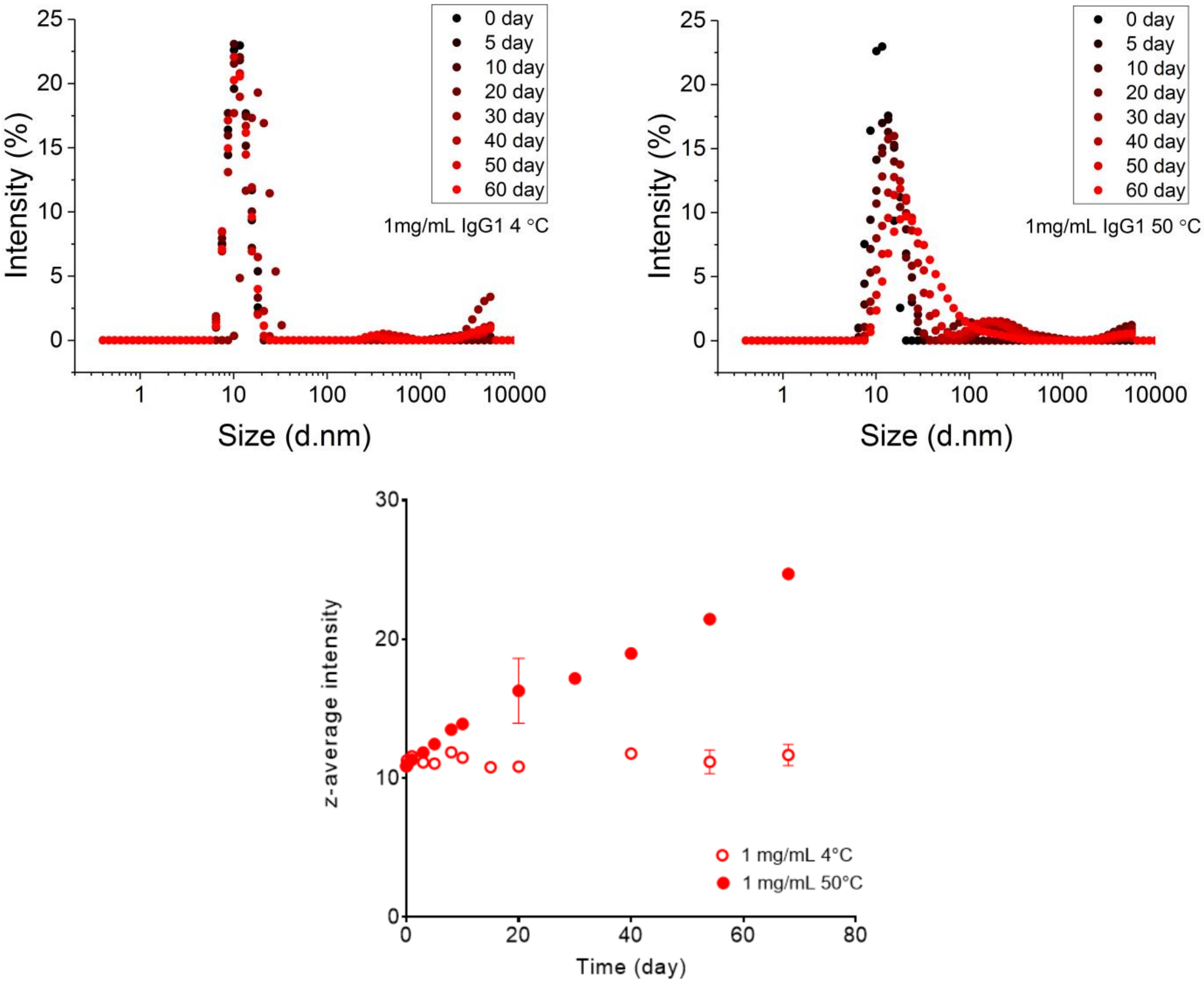

FIGURE S2: Dynamic light scattering of IgG1. The data of different incubation time are shown from black (0 day) to red (60 days). The z-average intensity of the major peak for IgG1 at 4 °C and 50 °C is shown over the experiment.

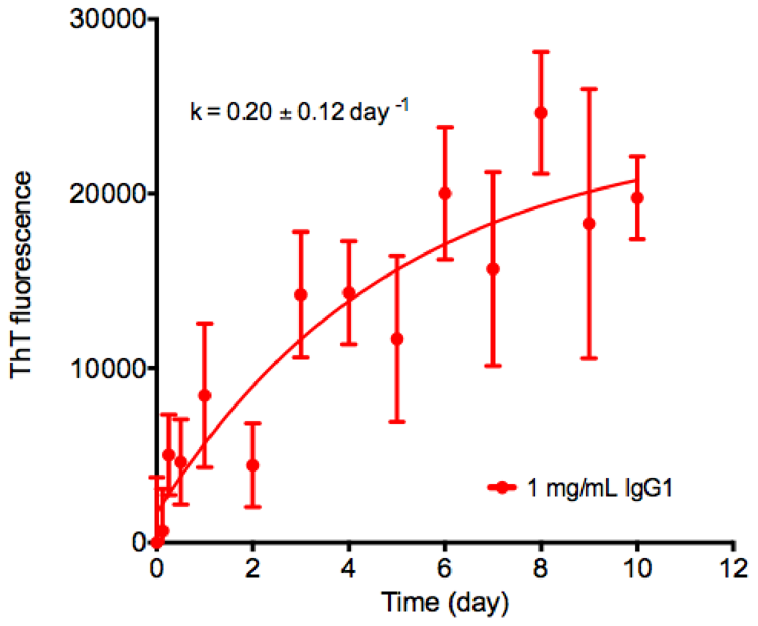

FIGURE S3: ThT assay of the IgG1 aggregation at 50 °C. The curve represent the single exponential fit to obtain the rate constant, *k*.

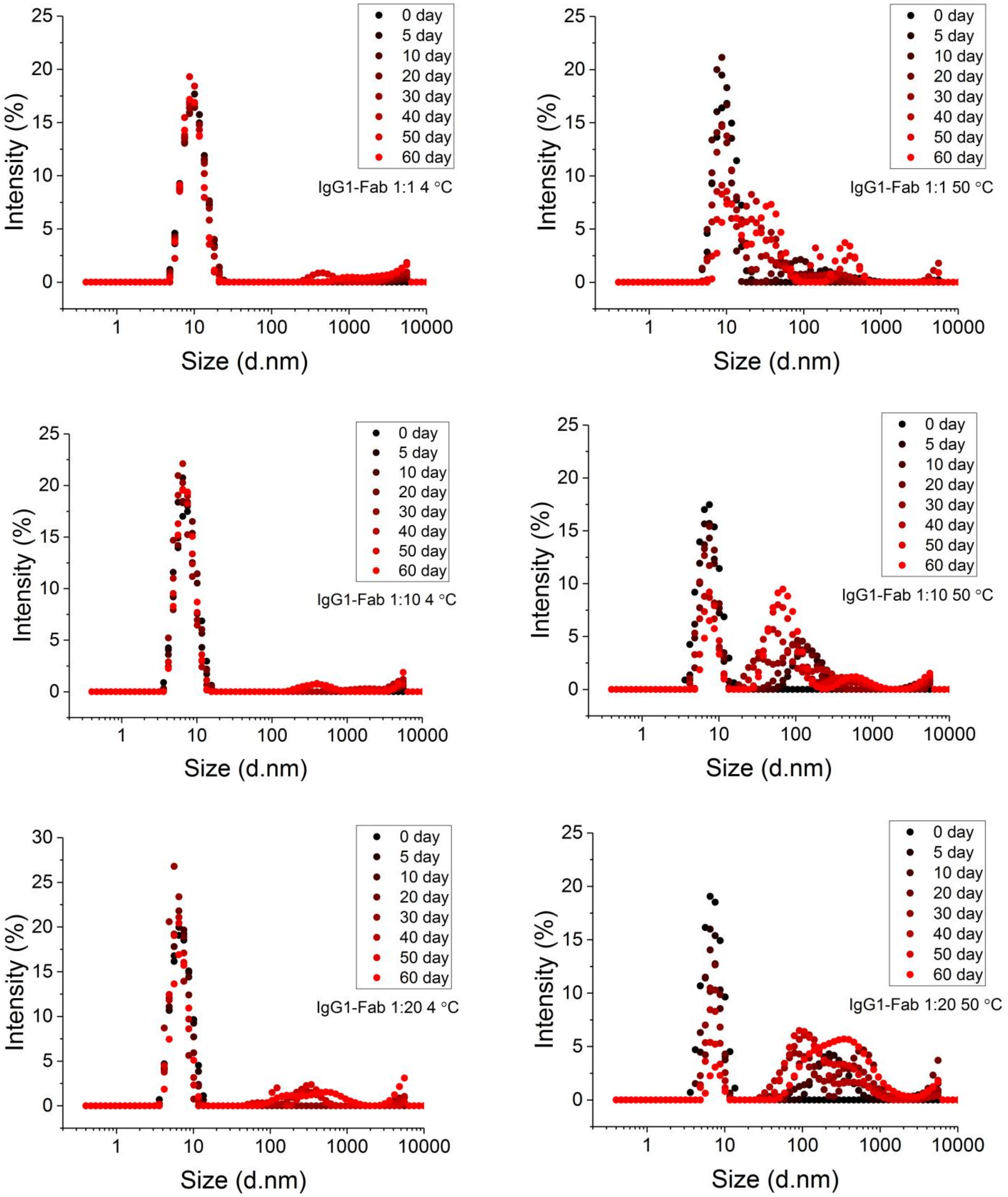

Intensity (%)
Size (d.nm)
0 day
5 day
10 day
20 day
30 day
40 day
50 day
60 day
IgG1-Fab 1:1 4 °C
IgG1-Fab 1:1 50 °C
IgG1-Fab 1:10 4 °C
IgG1-Fab 1:10 50 °C
IgG1-Fab 1:20 4 °C
IgG1-Fab 1:20 50 °C

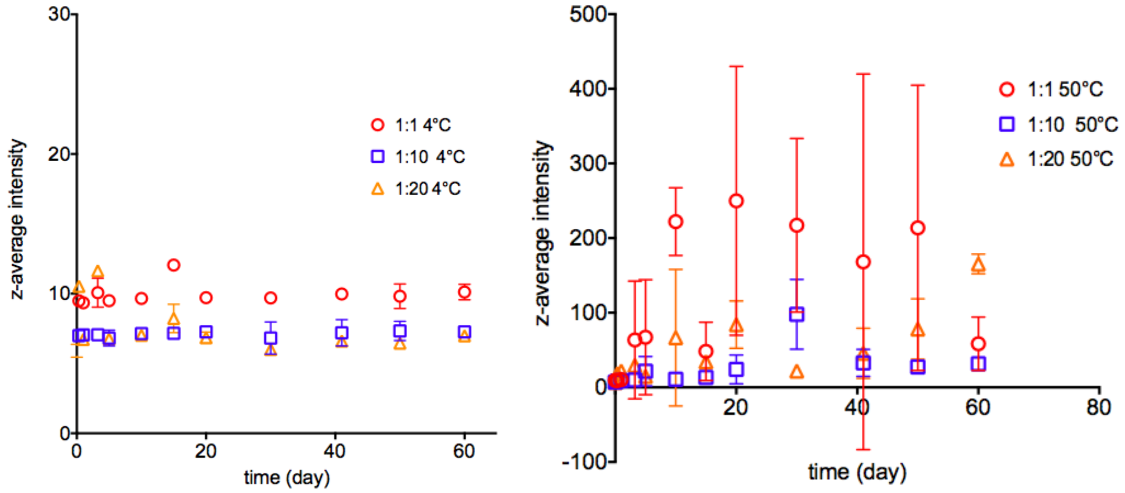

FIGURE S4: Dynamic light scattering of IgG1-Fab coformulations. The data of different incubation time are shown from black (0 day) to red (60 days). The z-average intensity of the major peak for IgG1-Fab at 4 °C and 50 °C over the experiment are shown for 1:1, 1:10 and 1:20 coformulations, respectively. The z-average intensity at 50 °C is very noisy due to the high polydispersity of the DLS data.

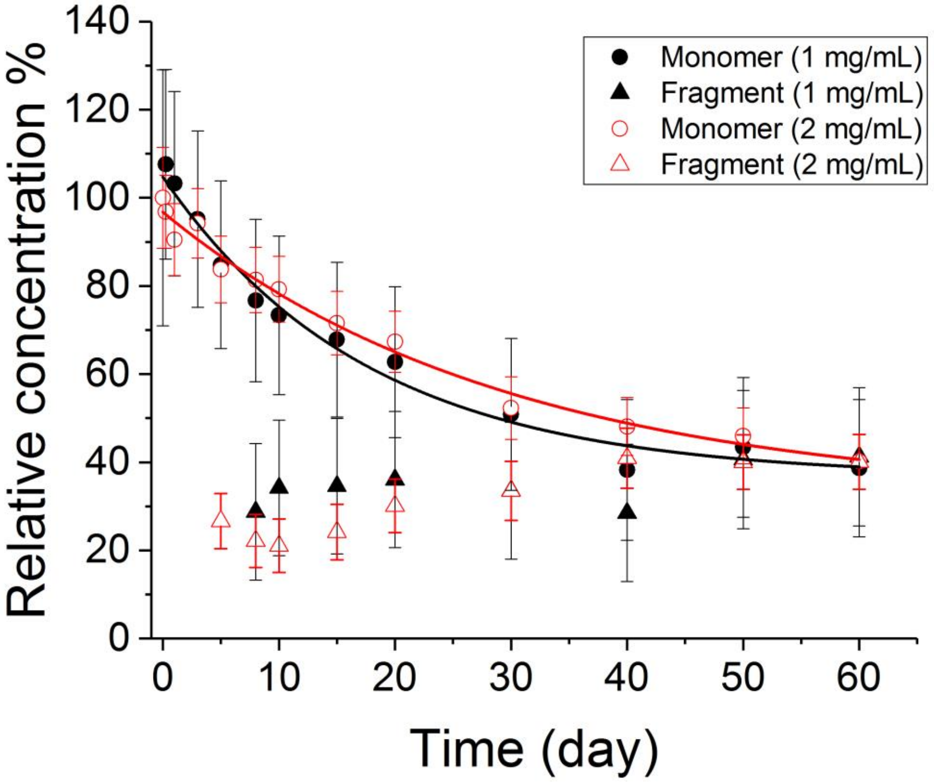

Figure S5: Degradation kinetics of IgG1 at 2 mg/mL 50 °C, pH 4.5. The data of 1 mg/mL is taken from Figure 4 and shown here as a reference. The change in monomer for 1 and 2 mg/mL are fitted to a single-exponential decay equation.

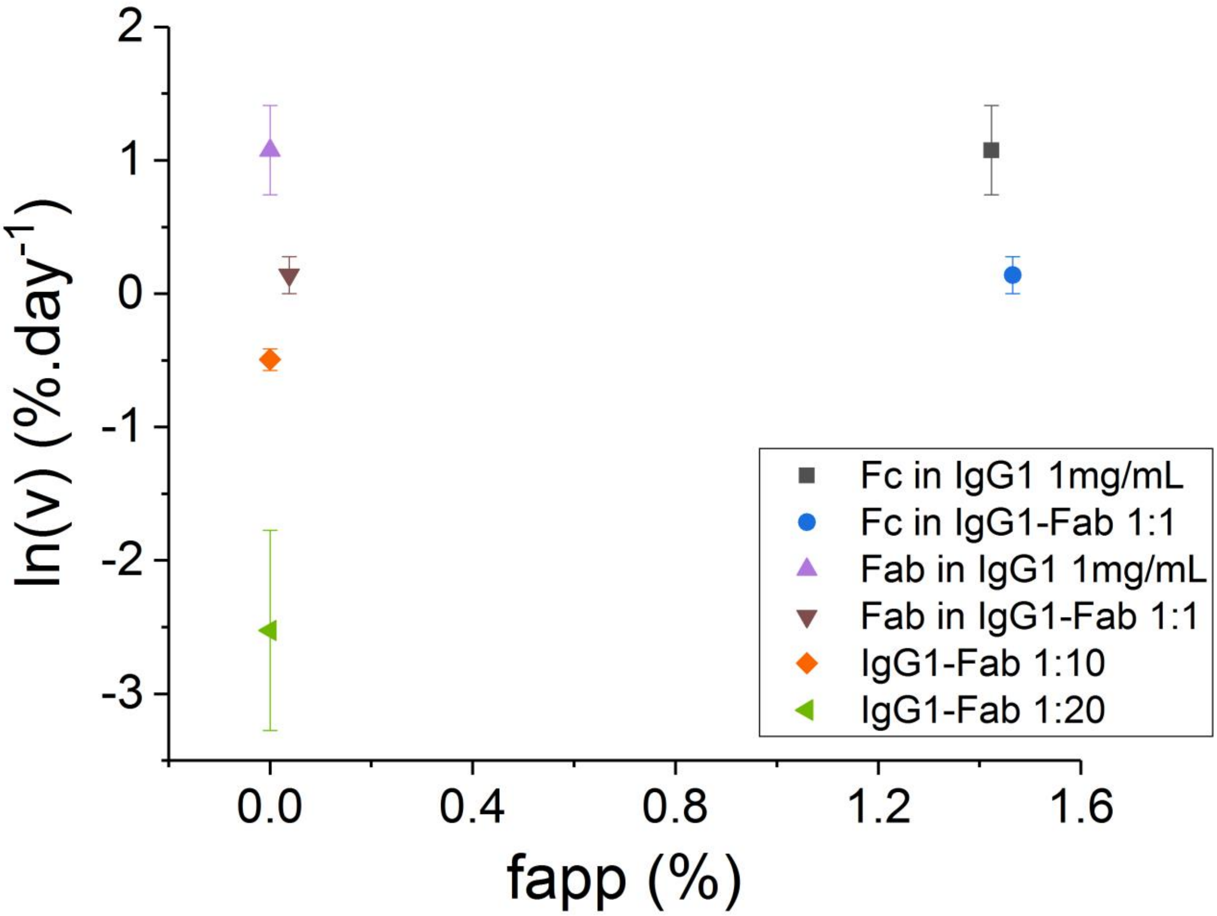

FIGURE S6: Correlation of the initial rates for monomer loss and the apparent fraction of unfolded (f_{app}, %).
